# Pulsed Electric Field Processing of Red Grapes (cv. Rondinella): Modifications of Phenolic Fraction and Effects on Wine Evolution

**DOI:** 10.3390/foods9040414

**Published:** 2020-04-02

**Authors:** Piergiorgio Comuzzo, Sabrina Voce, Cristian Grazioli, Franco Tubaro, Marco Marconi, Gianmaria Zanella, Marco Querzè

**Affiliations:** 1 Department of Agricultural, Food, Environmental and Animal Sciences, University of Udine, via Sondrio 2/A, 33100 Udine, Italy; voce.sabrina@spes.uniud.it (S.V.); cristian.grazioli@uniud.it (C.G.); franco.tubaro@uniud.it (F.T.); 2 JU.CLA.S. S.r.l., Vason Group, via Mirandola 49/A, 37026 Settimo di Pescantina (VR), Italy; marco.marconi@vason.it; 3 Enologica Vason S.p.A., Vason Group, via Nassar 37, 37029 San Pietro in Cariano (VR), Italy; gianmaria.zanella@vason.it; 4 Alintel S.r.l., via Mascarino 12/N, 40066 Pieve di Cento (BO), Italy; commerciale@alintel.it

**Keywords:** PEF, grape processing, red winemaking, maceration, wine evolution, aging, anthocyanins, vitisin A, tannins, wine color

## Abstract

Pulsed electric field (PEF) is a non-thermal technology able to promote color and polyphenols extraction from grape skins. Most of the publications about PEF in winemaking report data concerning international varieties, poorly considering minor cultivars and the medium/long-term effects of the treatment on wine composition during storage. PEF was applied at different specific energies (2, 10, and 20 kJ kg^−1^) on grapes of the low-color red cv. Rondinella, after crushing-destemming. Pressing yield, the evolution of color, and total phenolic index (TPI) were measured during skin maceration. Moreover, the wines were characterized for basic compositional parameters, color, anthocyanin profile, phenolic composition (glories indices), metal content (Fe, Cr, and Ni), and sensory characters, two and twelve months after the processing, in comparison with untreated samples and pectolytic enzymes (PE). PEF did not affect fermentation evolution, nor did it modify wine basic composition or metal content. Treatments at 10 and 20 kJ kg^−1^ led to higher color and TPI in wines, in comparison to PE, because of increased content of anthocyanins and tannins. The sensory evaluation confirmed these findings. Modifications remained stable in wines after twelve months. Glories indices and vitisin A content highlighted greater potential stability of wine color in PEF-treated wines.

## 1. Introduction

Color is one of the most important attributes for defining red wine quality. Its intensity and stability are related to phenolic compounds, in particular, anthocyanins. These molecules are located in the vacuoles of grape skin cells, and the red-purple or red-orange hue they confer to the wines is due not only to their nature and concentration but also to their transformation during maceration and fermentation, as well as to the reactions (i.e., polymerization, co-pigmentation, and condensation) they undergo during wine aging [1,2,3].

Certain grape varieties are characterized by a low anthocyanin content, and the production of wines from such low-colored grapes may require color correction practices, such as the blending with high-anthocyanin containing wines or the use of concentrated red musts, reducing the varietal fingerprint of the products obtained.

Aside from the various forms of mechanical intervention, a common technological tool for improving the extraction of color during skin maceration consists of the use of pectolytic enzymes (PE); this practice is able to increase the extraction of phenolic compounds, allowing to reduce contact time and improve wine color intensity and stability [4,5].

During the last decades, different emerging non-thermal technologies have been proposed, alternatively to traditional methods, for improving color extraction in red winemaking. Pulsed electric field (PEF) is a promising non-classical method, consisting of the application of short pulses (micro- to milliseconds) of high-voltage electric current (10–80 kV cm^−1^) to solid or liquid food products [6]. The mechanism through which PEF acts on organic tissues consists of the formation of pores on cell membranes (electroporation); this causes modifications of cell permeability, up to permanent cell breakdown [7].

In the food industry, PEF has been tested successfully for microbial inactivation (cold pasteurization) [8,9], as well as for increasing juice extraction yield or for the recovery of bioactive compounds from plant materials [10,11]. In winemaking, the potential of PEF has been exploited for improving the extraction of phenolic compounds from grape by-products [12], for the quality improvement of aged wines [13,14], for reducing wine spoilage yeasts and bacteria [15], as well as for inducing yeast autolysis [16] and accelerating wine aging on lees [17,18].

However, the most studied application of PEF in the wine industry has been their use for reducing maceration time in red wine processing. PEF has been able to increase the release of phenolic compounds from grape skins [19], improving anthocyanin content and wine color intensity [20]. PEF processing has allowed obtaining red wines with higher color intensities with respect to enzymatic treatments (PE) [21,22], whereas chemical composition (i.e., acidity, alcohol, pH), sensory attributes, and overall quality have seemed to be not influenced by PEF treatments [23,24].

Despite the several positive effects of the application of PEF in food and wine processing, this technology might be potentially harmful to the consumers due to the possible release of metals (especially iron, chromium, and nickel) from stainless steel electrodes. The risk is related to electrochemical reactions occurring at the electrode surface, provoking their corrosion [25,26]. Recent experiments [27] have reported a limited risk in the conditions used for PEF application in winemaking, but further investigations are needed in different experimental settings to guarantee the safety of the consumers.

This work aimed at evaluating the efficacy of PEF pre-treatment on the grapes of the low-color red cultivar Rondinella (*V. vinifera*), an Italian variety used for the production of Amarone della Valpolicella. The evolution of color and total polyphenols were studied during skin maceration, but also after two and twelve months since the end of alcoholic fermentation, for assessing if the compositional changes induced by PEF were stable over medium-long storage time. Besides basic analytical parameters (e.g., sugars, total acidity, pH, alcoholic strength, color indices), anthocyanin profile, some indices related to the composition of the phenolic fraction, metal content (Ni, Fe, and Cr), and the modifications induced on wine sensory characters were also considered, in comparison with wines produced from untreated grapes or by using extraction (pectolytic) enzymes.

## 2. Materials and Methods

### 2.1. Reagents and Materials

Sodium bisulfite, 96% (*v*/*v*) ethanol, ACS-grade hydrochloric acid (37%), reagent grade potassium metabisulfite, tartaric acid, and sodium hydroxide were purchased from Carlo Erba Reagents (Milan, Italy). HPLC-grade methanol, formic acid (98–100%), *n*-butanol, and iron(III)sulfate heptahydrate were from Sigma-Aldrich (St. Louis, MO, USA). Multielement (Al, Ag, As, B, Ba, Be, Bi, Ca, Cd, Co, Cr, Cu, Fe, K, Li, Mg, Ti, Tl, V, Zn) ICP standard solution 1000 µg/L in 5% HNO_3_ was from VWR (Radnor, PA, USA). Suprapur HNO_3_ 65% w/w and Suprapur H_2_O_2_ 30% w/w were from Merck (Darmstadt, Germany). Distilled water was purified with an Elgastat^®^ UHQ-PS system (Elga, High Wycombe, UK). All the chemicals employed were of analytical reagent-grade quality and were used as received.

Finally, the active dry yeast strain (Flavor 2000), the pectolytic enzyme preparation (Zimared Plus), the yeast nutrient supplement (V-Starter Premium), and the potassium metabisulfite used in winemaking protocols were supplied by Enologica Vason S.p.A. (S. Pietro in Cariano, VR, Italy).

### 2.2. PEF Treatments

Two hundred kilograms of crushed-destemmed Rondinella grapes were provided by a local winery (Valpolicella, Verona, Italy). The mash was sulfited with 150 mg·kg^−1^ of potassium metabisulfite and subjected to PEF treatment using the same pilot plant described by Comuzzo and colleagues [28].

PEF equipment was an 8 kV/30 A PEF generator (Model H.V.18kV_30A_Alintel Generator) coupled with a 100 × 30 mm i.d. poly(methyl methacrylate) (PMMA) cylindrical cell provided with two toroidal stainless steel electrodes. Both the cell and the generator were supplied by Alintel S.r.l. (Pieve di Cento, BO, Italy). The mash was pumped into the treatment cell by a single-screw volumetric pump (Liverani, Lugo RA, Italy) at a flow rate of 250 L h^−1^. The electric field was applied at 1.5 kV cm^−1^ with single pulse durations of 0 μs (no pulse, Untreated), 1 μs (corresponding to a total specific energy of 2 kJ kg^−1^—PEF 2), 5 μs (total specific energy 10 kJ kg^−1^—PEF 10), and 10 μs (total specific energy 20 kJ kg^−1^—PEF 20). The generator produced square-wave pulses at a frequency of 400 Hz.

Besides PEF treatments and untreated samples, an additional aliquot of the latter (no pulse) was collected at a pilot plant outlet and immediately supplemented with 20 mg kg^−1^ of pectolytic enzyme preparation (ENZ). All experiments were performed in three repetitions (8 kg each) at room temperature (22 °C), and the temperature increase of the mash after treatments was lower than 3 °C. All samples were collected in 10 L volume food-grade polyethylene terephthalate (PET) vessels and further processed as detailed below.

### 2.3. Winemaking Protocols

PEF-processed, enzyme-treated, and untreated mash were immediately supplemented with 200 mg L^−1^ of active dry yeasts (prepared according to the supplier instructions) and 200 mg L^−1^ of yeast nutrient preparation. Skin maceration was carried out at 24 °C, monitoring color parameters (Abs 420, 520, and 620 nm) and total phenolic index (TPI) daily. The pomace was punched-down twice per day for ensuring homogenization and aeration of the mash; in addition, on the second day of fermentation, all samples were oxygenated by injecting 150 mL L^−1^ of air into the pomace, using a stainless steel microporous diffuser.

Draining and pressing were carried out after seven days of maceration. All samples were subjected to two pressing cycles at a maximum pressure of 2 bar each, using a water press (Model W80, Grifo Marchetti, Piadena, CR, Italy). Fermentation continued until complete sugar depletion.

At the end of alcoholic fermentation, wines were racked, sulfited with 60 mg L^−1^ of potassium metabisulfite, and kept at 0/+4 °C for five days, allowing tartaric stabilization. After bottling and sealing with crown cap closures, samples were stored at 20 °C until analysis, carried out after two and twelve months of bottle storage.

### 2.4. Analytical Determinations

#### 2.4.1. Pressing Yield

The juice extraction yield was evaluated, after pressing, as the percentage ratio between the weight of the juice and that of the mash, as suggested by Praporsic and co-workers [29].

#### 2.4.2. FTIR Analysis

Basic quality control parameters were measured on wines, two and twelve months after the processing, using an FTIR spectrometer model Winescan™ FT-120 (FOSS, Hillerød, Denmark). Alcoholic strength, reducing sugars, total acidity, volatile acidity, pH, malic, lactic, and citric acid, total dry extract, glycerol, potassium, and ash were determined. Data were mean values of two repeated measurements carried out on each replicated sample.

#### 2.4.3. Wine Color Parameters and TPI

Color intensity (CI), color hue (CH), and TPI were determined on wine samples and during skin contact using a V-530 UV-Vis spectrophotometer (Jasco Co. Ltd., Tokyo, Japan). CI was calculated by the following equation:

(1) $$\mathrm{CI}\;=\left({\mathrm{Abs}}_{420nm}+{\mathrm{Abs}\;}_{520nm}+{\mathrm{Abs}}_{620nm}\right)\times 10$$

CH was expressed as the ratio between Abs 420 nm and Abs 520 nm. Absorbances were measured in 1 mm optical path-length glass cuvettes (Hellma Analytics, Mülheim, Germany), without dilution and reading against distilled water. All samples were filtered on 0.8 µm pore size nylon membranes (Minisart, Sartorius, Göttingen, Germany) before analysis.

TPI was determined as follows: 1 mL of filtered must or wine was diluted in a 50 mL volumetric flask; absorbance was measured at 280 nm against distilled water in disposable 10 mm path-length UV-grade PMMA cuvettes (Kartell S.p.A.—Labware Division, Noviglio, MI, Italy). TPI was calculated multiplying the Abs 280 nm by the number of dilutions (*n* = 50).

#### 2.4.4. Anthocyanins

Anthocyanins were determined on wines, after two and twelve months of storage, by spectrophotometry and by RP-HPLC. The spectrophotometric determination was carried out according to the procedure described by Ribéreau-Gayon and Stonestreet [30], based on the ability of bisulfite ion to bleach anthocyanins.

RP-HPLC separation, instead, was performed by the method reported by Morata et al. [31], with the following modifications. The column was a Zorbax Ecliplse Plus C_18_ (4.6 × 150 mm, 5 µm particle size), purchased from Agilent Technologies (Santa Clara, CA, USA) and conditioned at 25 °C. The HPLC system was an LC-2010 AHT liquid chromatograph (Shimadzu, Kyoto, Japan) equipped with an integrated autosampler and UV–visible detector; detection was performed at 525 nm, and the solvent flow rate was set at 1.2 mL·min^−1^.

The peaks related to delphinidin-3-*O*-glucoside (D3G), cyanidin-3-*O*-glucoside (C3G), petunidin-3-*O*-glucoside (Pt3G), peonidin-3-*O*-glucoside (Peo3G), malvidin-3-*O*-glucoside (M3G), malvidin-3-*O*-(6-acetyl)-glucoside (M3GAc), malvidin-3-*O*-(6-*p*-coumaroyl)-glucoside (M3GpCm), carboxypyranomalvidin-3-*O*-glucoside (vitisin A—VitA), and pyranomalvidin-3-*O*-glucoside (vitisin —VitB) were tentatively identified by comparing their retention times and chromatographic profile with the literature data [32,33]. Absolute areas of the detected peaks were used for the elaboration of the data.

#### 2.4.5. Total Tannins

Total tannins (proanthocyanidins) were measured on wines (two and twelve months of storage) by the classic methodology developed by Bate-Smith [34]. Briefly, 2 mL of wine, previously diluted 1:50 in distilled water, was added to 6 mL of Bate-Smith reagent (150 g L^−1^ of iron(III)sulfate in 1:1 *v*/*v* *n*-butanol: 37% hydrochloric acid solution). An aliquot of approximately 3 mL of this mixture was heated at 100 °C for 30 min in Pyrex test tubes, while the remaining portion was stored in the dark for the same time. After cooling of the heated tubes, the absorbance of both samples was measured at 550 nm, using 10 mm disposable PMMA cuvettes (Kartell S.p.A.—Labware Division, Noviglio, MI, Italy). Total tannins (TT) in g L^−1^ were calculated on the basis of the following equation.

(2) $$\mathrm{TT}\;=\left(Abs_{550\;nm}^{heat}-Abs_{550\;nm}^{dark}\right)\times 0.1736\times 50$$

#### 2.4.6. Glories Indices

These indices allow an overview of the state and the evolution of wine polyphenolic fraction. In the present study, three indices were determined on wines, after two and twelve months of storage: index of hydrochloric acid (IHCl), index of ethanol (IEtOH), and index of polymerized pigments (IPP) were analyzed according to the procedures described by Glories [35].

The former (IHCl) was associated with the degree of polymerization of tannins; 10 mL of wine was mixed with 5 mL of distilled water and 15 mL of 37% hydrochloric acid (HCl). Acid addition promotes the precipitation of condensed tannins, determining a reduction of the absorbance at 280 nm. The mixture was stored in the dark at room temperature for 7 h and then centrifuged (2000 rpm for 5 min). The supernatant was diluted 1:25, and the absorbance was measured at 280 nm (d7h); IHCl is the percent diminution of Abs 280 nm, with respect to the value measured for the initial mixture (d0h) in the same conditions (25 dilutions).

(3) $$\mathrm{IHCl}\;=\frac{d0h-d7h}{d0h}\times 100$$

IEtOH represents the polyphenolic fraction bound to polysaccharides (PS). Ethanol promotes the precipitation of PS and the bound polyphenols, provoking a decrease of the Abs 280 nm. The assay consisted of mixing 1 mL of wine and 9 mL of 96% (*v*/*v*) ethanol. Samples were stored in the dark at room temperature for 24 h and then centrifuged. The supernatant was collected, diluted 10 times in distilled water, and Abs 280 nm was measured (d24h). IEtOH was determined with the following equation, where d0h is the absorbance of the initial mixture, also diluted 10 times.

(4) $$\mathrm{IEtOH}\;=\frac{d0h-d24h}{d0h}\times 100$$

IPP is indicative of the state of combination and stability of anthocyanins; a higher degree of combination makes anthocyanins less prone towards sulfite bleaching [35]. IPP was determined by preparing two test tubes; in the former, 1 mL of wine was mixed with 40 µL of distilled water and 9 mL of hydroalcoholic-tartaric buffer (5 g L^−1^ tartaric acid and 12% *v*/*v* ethanol, buffered at pH 3.20 with 10 M sodium hydroxide). The latter was prepared with the same modalities, but replacing distilled water with 40 µL of sodium metabisulfite solution (20% w/v). After 5 min, absorbance was read at 420 and 520 nm for both tubes. IPP was calculated by the following equation.

(5) $$\mathrm{IPP}\;=\frac{Abs\;420_{SO2}+Abs\;520_{SO2}}{Abs\;420_{H2O}+Abs\;520_{H2O}}\times 100$$

For all three indices, absorbances were measured in 10 mm disposable PMMA cuvettes (Kartell S.p.A.—Labware Division, Noviglio, MI, Italy) against distilled water.

#### 2.4.7. Metal Analysis

To assess the eventual release of metals from the electrodes during PEF processing, the concentrations of iron (Fe), nickel (Ni), and chromium (Cr) in wines were analyzed by inductively coupled plasma spectrometry (ICP).

All samples were prepared for ICP analysis by microwave wet mineralization. Complete mineralization of samples was obtained by an MLS-1200 MEGA microwave digester (Milestone-FKV, Bergamo, Italy) equipped with an EM-45 exhauster of nitric acid fumes, a control panel, and an MDR-1000/6/100/110 rotor in polypropylene, capable of holding up to six digestion vessels. Wine samples were carefully homogenized, and 1 mL was added to a mixture of 2 mL ultrapure-grade HNO_3_ and 0.5 mL ultrapure-grade H_2_O_2_. The resulting solutions were digested by microwave, and, after cooling, they were diluted to 10 mL with ultrapure water to be analyzed via ICP-MS. The internal standard (rhodium, 100 µg/L) was subsequently added to the samples before the ICP-MS analysis. This kind of sample pre-treatment was selected instead of dilution to ensure an accurate element determination by ICP-MS, reducing the interferences due to the high content of organic compounds in wine [36,37].

ICP-MS analysis of the sample was performed with a Nexion 350X instrument (Perkin Elmer, Waltham, MA, USA) equipped with collision cell and KED (Kinetic Energy Discrimination) for polyatomic isobaric interferences reduction.

Stock standard solutions for metal analysis calibration were obtained by dilution of the corresponding standard mother solutions 1000 µg/L with ultrapure water and adding ultrapure-grade 65% w/w HNO_3_ to achieve a final concentration of 0.5%. The concentrations achieved for each element were as follows: 0, 1, 5, 10, 20, 50, and 100 µg/L.

#### 2.4.8. Sensory Evaluation

A blind sensory analysis of wines was carried out after two and twelve months of storage by a ranking test. The samples were labeled with three-digit numerical codes and submitted (according to a randomized service order) to the evaluation of a panel composed of seven selected subjects (three females and four males, all enologists, age 25–45 years). Each panelist was asked to evaluate the wines based on a series of pre-established attributes, ranking them in the order of intensity. Attributes were color intensity, orange hue, (fruity) aroma intensity, vegetal/herbaceous notes, body/structure, astringency, and global impression.

### 2.5. Statistical Analysis

Data were means and standard deviations (SD) of three measurements, originated from three replicated experiments. For chemical analysis, one-way ANOVA was carried out, and significant differences were assessed by Tukey Honest Significant Difference (HSD) test at *p* < 0.05. The software used was Statistica for Windows, ver. 8.0 (Statsoft Inc. Tulsa, OK, USA).

Sensory analysis results were elaborated by the Friedman test, as described by Barillere and Benard [38], in order to evaluate the minimum significant difference between ranks (*p* < 0.05). If, for a given attribute, significant differences were found between the sums of the ranks calculated for the different samples (ΣR), the wine with the lowest ΣR was the one characterized by the greatest intensity for that attribute.

## 3. Results and Discussion

### 3.1. Effect of PEF Processing on Pressing Yield

PEF pre-treatment determined an increased pressing yield, in agreement with the literature [28,29,39]. The average pressing yield of the untreated sample (control) after maceration was 70.3% (w/w). This value increased to 79.7% w/w for enzyme-treated samples (+13.5% compared to the control), likely due to the capacity of pectolytic enzymes to degrade skin tissues. The less intense PEF treatment (2 kJ kg^−1^) led to an additional increase of yield: the average value estimated was 83.1% w/w (+18.3% with respect to the control). This value surprisingly decreased to 76.1% w/w (+8.2%) for the samples treated at 10 kJ kg^−1^, and to 73.3% w/w (+4.4%) for those processed at 20 kJ kg^−1^.

This inverse relationship between pressing yield and specific energy applied has been already observed in other experiments [28], and it might be explained by observing the aspect of the marc during skin maceration and after pressing. The marc of the PEF-treated samples showed a different consistency during maceration, with respect to untreated and enzyme-treated grapes. In particular, PEF-processed marc seemed characterized by thinner skins, and the integrity of skins themselves was less evident in PEF samples; this effect appeared more evident as the specific energy increased (Figure S1, Supplementary Material). In addition, after pressing, the PEF-processed marc remained more compact and humid. These observations might suggest that the greater degree of disintegration of the solid parts induced by PEF (Figure S1, Supplementary Material) might have reduced the draining of the marc during pressing, progressively decreasing the extraction yield, as the specific energy increased.

To the best of author’s knowledge, this effect was not reported in the literature, but it might have a non-negligible impact on the practical application of PEF technology in the wineries. In addition, it is well known that a higher mechanical degradation of grape skins may provoke higher turbidity and greater amounts of lees after pressing, as already reported for PEF processing in other experiments [28].

### 3.2. Effect of PEF Processing on the Extraction of Color and Phenolic Compounds during Maceration

**Figure 1:**
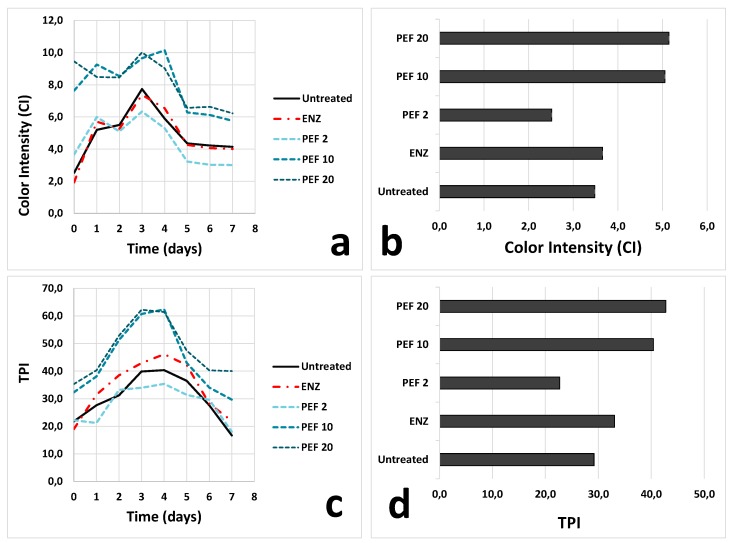
Trends measured for color intensity (**a**,**b**) and total phenolic index (TPI) (**c**,**d**) in the samples analyzed during skin maceration up to draining/pressing (day 7) (**a**,**c**) and at the end of alcoholic fermentation (day 9) (**b**,**d**). Untreated: control, no treatment; ENZ: pectolytic enzymes (20 mg kg^−1^); PEF 2: PEF treatment, 2 kJ kg^−1^; PEF 10: PEF treatment, 10 kJ kg^−1^; PEF 20: PEF treatment, 20 kJ kg^−1^. Figures a and c have been modified from Comuzzo et al. [40]. PEF, pulsed electric field.

As expected, PEF allowed a faster and greater release of color and phenolic compounds from grape skins (Figure 1). In particular, the trials performed at 10 and 20 kJ kg^−1^ determined an immediate extraction of color since the beginning of the maceration, leading, consequently, to a significantly higher CI at draining/pressing, with respect to the untreated and enzyme-treated controls (Figure 1a). This trend remained stable at the end of alcoholic fermentation, when the wines treated at 10 and 20 kJ kg^−1^ showed a CI value of approximately 30% greater than in untreated and ENZ products (Figure 1b).

TPI was also higher in PEF 10 and PEF 20 macerations; the latter showed the most abundant polyphenol content at draining (Figure 1c). As observed for CI, the differences found for TPI also remained stable up to the end of alcoholic fermentation (Figure 1d).

It was interesting to observe that, when applied at suitable intensity (e.g., specific energy 10–20 kJ kg^−1^), PEF processing was able to notably increase the limited color and phenolic contents of untreated Rondinella grapes (CI = 3.5 and TPI = 29.2 at the end of alcoholic fermentation), increasing them to more acceptable values (CI > 5.0; TPI > 40.0).

Surprisingly, the grapes processed at 2 kJ kg^−1^ exhibited a low color and polyphenol extraction, and both the maceration curves and the values detected at the end of fermentation were even lower than those found in untreated samples.

This behavior might have certain similarities with previous data obtained by applying PEF pre-treatment on white grapes [28]. The authors observed that the samples treated at the lowest specific energy (11 kJ kg^−1^) were subjected to a more intense browning than those processed at 20 kJ kg^−1^. They explained this fact by hypothesizing that the lower duration of the pulse, in the treatments with low specific energy, might promote the release of water and small molecules from skin cells, playing a potential role in determining a higher pressing yield, as observed for PEF 2 samples (Section 3.1). In such conditions, however, the low molecular weight phenolic compounds released might be easily oxidized as they are extracted, because the reactivity of flavanols towards oxidation is higher for the small molecules, while it decreases as the molecular complexity increases (degree of polymerization, glycosylation) [41].

Contrary, when the specific energy and the intensity of the PEF treatment increase (e.g., PEF 10 and PEF 20), electroporation creates larger pores in cell membranes [42], potentially favoring the release of large and more complex phenolic molecules, which may protect color compounds (e.g., anthocyanins) towards oxidation [43].

**Figure 2:**
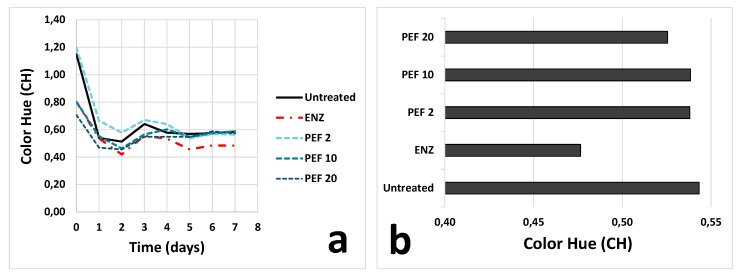
Trend measured for color hue in the samples analyzed during skin maceration up to draining/pressing (day 7) (**a**) and at the end of alcoholic fermentation (day 9) (**b**). Refer to Figure 1 for sample codes.

Color hue (CH) might partially confirm these hypotheses; the curve collected for PEF 2 grapes during maceration was above those obtained for the other samples (Figure 2a), highlighting an extracted coloring fraction characterized by more intense orange nuances. This effect was no more evident since the fifth day of maceration. Finally, another interesting observation concerning Figure 2b was that PEF pre-treatment led to wines characterized by less red and more orange hues (higher CH), with respect to pectolytic enzymes.

### 3.3. Effect of PEF Processing on Wine Basic Composition and Metal Content

PEF pre-treatment of the grapes did not affect fermentation evolution, nor did it modify wine basic composition in the twelve months after the treatment (Table 1). Minor differences were found concerning total dry extract (TDE), which was higher in ENZ wines and in PEF 10 and PEF 20 samples, confirming the extraction trend observed during maceration (Figure 1). PEF 2 wines showed the lowest TDE value, resulting even lower than that determined for untreated and reinforcing the hypothesis described in Section 3.2.

Another interesting difference among samples concerned the acidic fraction. Malolactic fermentation (MLF) occurred to some extent in all wines, but the data reported in Table 1 show that the consumption of malic acid was more intense in some of the treated wines (both ENZ and PEF) than in untreated. The diminution of malic acid concentration over twelve months of storage also affected total acidity and pH; however, while the final values detected for these two parameters in ENZ and PEF 2 were similar to those of untreated wine, in PEF 10 and PEF 20, a slightly lower total acidity and slightly higher pH value were evident (Table 1). The reason for the more intense evolution of MLF in the wines obtained from ENZ and PEF-treated grapes might be connected to greater extraction of nutrients and survival factors (useful for bacteria growth) from grape skins. These aspects were not considered in the present study; however, the investigation of the eventual effects of PEF pre-treatment on malolactic fermentation enhancement might be an interesting topic to be developed in future experiments.

Table 2 reports the concentrations detected for iron, nickel, and chromium in wines after twelve months of storage. Despite different papers investigated the possible corrosion of the electrodes and the subsequent release of metals in PEF-treated foods (see Section 1), only a limited number of them report data concerning wine [27]. The values shown in Table 2 confirm such results: no significant release of iron, chromium, and nickel was induced by PEF in the conditions of the experiment. While the concentrations found for chromium had the same order of magnitude of those reported in the reference cited above [27], the levels of iron and nickel detected in the present study were significantly lower, probably because of the fact that the experimental vinifications, described in Section 2.3, were carried out in polyethylene and glass containers, avoiding stainless steel.

### 3.4. Effect of PEF on Wine Color and the Composition of Phenolic Fraction

The trend observed during skin maceration and at the end of alcoholic fermentation (Figure 1) remained evident after two months of storage (Table 3a). Color intensity, TPI, anthocyanins, and total tannins were significantly higher in PEF 10 and PEF 20 samples than in untreated wines. Enzyme assisted extraction showed an intermediate behavior, while PEF 2 had the lowest concentration of color and phenolic compounds. This tendency remained evident also after twelve months, even if a slight CI diminution, more intense in PEF 20 samples, was highlighted over time (Table 3b). PEF pre-treatment allowed an average gain in anthocyanins and tannin concentration, for the low-color variety Rondinella, of approx. 60 and 900 mg L^−1^, respectively, in two months of storage.

Color hue increased in all wines over the storage period. Two months after the processing, similarly to what observed in Figure 2b, ENZ maintained the lowest CH and, consequently, a color characterized by more intense purple/red nuances. These differences were evened after twelve months when wines showed only slight differences regarding CH.

Glories indices might give other interesting indications about the state of a combination of the phenolic fraction. After two months (Table 3a), IHCl was higher in ENZ, PEF 10, and PEF 20 samples, suggesting that PEF was able to extract greater amounts of polymerized polyphenols from grape skins, as already known for pectolytic enzymes. PEF 2 had a low IHCl, probably because of a less intense induced electroporation, as explained in Section 3.2. IEtOH also revealed a similar trend; this index gives an indication about the presence of tannin-polysaccharides complexes, and the higher values detected after two months of storage for PEF 10 and PEF 20, may potentially have a role in improving mouthfeel and roundness of the wines, as well as in reducing the sensory perception of astringency [35]. Surprisingly, enzymes had a poor effect on IEtOH values in the conditions tested.

The evolution of IHCl over storage time was evident comparing Table 3a,b. In the time interval between two and twelve months, this index increased in PEF 2 and untreated wine, but, in ENZ and the other two PEF-processed samples (PEF 10 and PEF 20), it remained fairly constant, with just a slight decrease of average values. This might suggest a faster evolution of the phenolic fraction in PEF 10, PEF 20, and ENZ. In these samples, in fact, polyphenols reached a greater degree of polymerization in a shorter period of time (two months), and the slight diminution of IHCl after twelve months might be related to the precipitation of the most polymerized fractions. All wines, indeed, showed a small accumulation of sediment on the bottom of the bottles at the end of the storage period. On the contrary, the evolution of untreated wines was slower, and their IHCl continued to increase during the whole period of observation (twelve months). Even if these findings allow only hypothesizing such potential role of PEF in favoring the evolution of wine phenolic fraction and further investigations should be carried out, the results of the present experiment are in agreement with those published by Zeng and colleagues, who observed that the application of high voltage electric fields might accelerate wine aging [44].

IPP was also affected by the different treatments. This parameter is an indicator of the degree of combination of anthocyanins with other molecules (e.g., flavanols), and the higher the value, the greater the stability of wine pigments [35]. After two months, IPP was greater in PEF-processed wines (Table 3a); PEF 2 and PEF 20 only slightly differed from untreated and ENZ samples, while PEF 10 showed a significant IPP increase (averagely, + 9 units with respect to Untreated). IPP further raised during storage for all wines, but on month twelve, PEF 10 remained the sample with the highest index value (Table 3b). The greater IPP found in PEF 10 samples might be reasonably connected with the elevated CI found in these wines at the end of the storage period (Table 3b).

The HPLC profile of anthocyanins allowed further considerations from this point of view. After a storage period of two months (Table 4a), the data recorded agreed with the other parameters collected for characterizing the phenolic fraction (Table 3a). The lowest concentration of anthocyanins was found for PEF 2 samples, while average values became progressively higher, passing from untreated to ENZ and, finally, to PEF 10 and PEF 20 wines. Interestingly, vitisin A was also more present in the latter (particularly in PEF 10), reflecting potentially higher color stability [31] and confirming the trend highlighted by IPP (Table 3a).

Anthocyanins were subjected to a significant decrease, passing from two to twelve months of storage (Table 4b), presumably due to oxidation phenomena and to their involvement in polymerization reactions (also leading to the increase of IPP discussed previously). M3G and Vit A were the two most important anthocyanins in the wines at the end of the period considered. Concerning the former, the highest concentrations were detected in PEF 10, while the latter was significantly more abundant in both the PEF samples obtained with the highest specific energies (10 and 20 kJ kg^−1^). Vitisin B (M3G acetaldehyde adduct) was not found in detectable amounts in any of the Rondinella wines analyzed in this study.

The highest presence of Vit A in the wines processed by PEF is an interesting perspective for the use of this technology in winemaking because of the elevated stability of such pigments and their relevant impact on the color of aged wines [31].

### 3.5. Effect of PEF on Wine Sensory Characters

The sums of the ranks (ΣR) calculated after the ranking test are reported in Table 5, together with the results of statistical elaboration (Friedman test). According to Barillere and Benard [38], if a significant difference is found between two samples for a given attribute, the wine in which that attribute was perceived with the greater intensity is the one with the lowest ΣR.

Sensory evaluation results confirmed the trends highlighted for wine color and the other analytical parameters. In fact, the wines obtained by PEF 10 and PEF 20 treatments were visually perceived as significantly more colored after two months (Table 5a), and such a higher color intensity was maintained for the whole storage period (Table 5b). Contrary, PEF 2 showed the lowest color intensity and the most intense orange hue at both the sampling points.

No relevant differences were highlighted for the other attributes evaluated. However, it was interesting to observe that PEF 10 and PEF 20 wines were perceived as averagely more full-bodied (Table 5a) and structured after two months, probably for the greater IHCl and IEtOH they had on that time, with respect to the other samples (Table 3a). Finally, PEF 2 received the lowest score, concerning global impression at the end of the storage period.

## 4. Conclusions

PEF pre-treatment of grapes allowed a significant and durable increase of the color intensity and stability for the wines of the low-color red cv. Rondinella. Treatments with a specific energy of 10–20 kJ kg^−1^ were the most performing, leading to higher concentrations of anthocyanins and tannins after one year of storage and greater amounts of polymerized pigments and vitisin A with respect to untreated grapes and the use of pectolytic (extraction) enzymes. Lower specific energies (2 kJ kg^−1^) gave positive results for their ability to increase pressing yield but had a negative impact on wine color and phenolic composition.

PEF confirmed to be an interesting and promising technology to be applied for the grape processing of low color varieties; sensory evaluations highlighted a significant gain in color for experimental wines. This potential of PEF technology might support winemakers in producing stable varietal wines, without needing to correct the color by blending with other (colored) varieties, preserving the peculiar sensory characteristics of their productions.

## Figures and Tables

**Table 1:** Basic analytical parameters (means ± SD) detected in the wines after twelve months of bottle storage.

**Sample Code ^1^**	**Alcoholic Strength** **(% *v*/*v*)**			**Reducing Sugars** **(g L^−1^)**			**Total Dry Extract** **(g L^−1^)**		
Untreated	11.87	±	0.01	2	±	0	21.3	±	0
ENZ	12.01	±	0.03	3	±	0	22.2	±	0.1
PEF 2	12.37	±	0.01	2	±	0	20.2	±	0
PEF 10	12.00	±	0.02	2	±	0	22.5	±	0
PEF 20	12.05	±	0.02	2	±	0	22.8	±	0
**Sample Code**	**Total Acidity** **(g L^−1^)**			**Volatile Acidity** **(g L^−1^)**			**pH**		
Untreated	5.32	±	0.02	0.43	±	0.01	3.41	±	0
ENZ	5.14	±	0.03	0.42	±	0	3.38	±	0.01
PEF 2	5.12	±	0.03	0.42	±	0.01	3.41	±	0.01
PEF 10	5.02	±	0.03	0.39	±	0.01	3.45	±	0.01
PEF 20	4.90	±	0.01	0.45	±	0.01	3.45	±	0
**Sample Code**	**Malic Acid** **(g L^−1^)**			**Lactic Acid** **(g L^−1^)**			**Citric Acid** **(g L^−1^)**		
Untreated	0.67	±	0.02	1.05	±	0.04	0.25	±	0.01
ENZ	0.34	±	0.02	1.18	±	0.05	0.24	±	0.01
PEF 2	0.40	±	0.02	1.25	±	0.01	0.28	±	0.01
PEF 10	0.58	±	0.02	1.05	±	0.01	0.27	±	0
PEF 20	0.29	±	0.04	1.23	±	0.03	0.25	±	0
**Sample Code**	**Glycerol** **(g L^−1^)**			**Potassium** **(g L^−1^)**			**Ash** **(g L^−1^)**		
Untreated	8.4	±	0	0.8	±	0	1.8	±	0
ENZ	8.2	±	0.1	0.8	±	0	1.7	±	0
PEF 2	8.5	±	0	0.8	±	0	1.6	±	0
PEF 10	8.5	±	0	0.8	±	0	1.9	±	0
PEF 20	8.8	±	0	0.8	±	0	1.8	±	0

^1^ Untreated: control, no treatment; ENZ: pectolytic enzymes (20 mg kg^−1^); PEF 2: PEF treatment, 2 kJ kg^−1^; PEF 10: PEF treatment, 10 kJ kg^−1^; PEF 20: PEF treatment, 20 kJ kg^−1^. PEF, pulsed electric field.

**Table 2:** Concentrations (means ± SD) of chromium, iron, and nickel detected in the wines after twelve months of bottle storage. The values are expressed in µg L^−1^. Different letters mark significant differences according to ANOVA and Tukey HSD test (*p* < 0.05). Refer to Table 1 for sample codes.

	Chromium (^52^Cr)				Total Iron (^56^Fe + ^57^Fe)				Total Nickel (^58^Ni + ^60^Ni)			
Untreated	33	±	34	a	1080	±	517	a	49	±	30	a
ENZ	8	±	8	a	1092	±	144	a	14	±	2	a
PEF 2	13	±	14	a	909	±	263	a	19	±	3	a
PEF 10	10	±	0	a	1197	±	0	a	14	±	0	a
PEF 20	2	±	1	a	752	±	63	a	24	±	12	a

**Table 3:** Values (means ± SD) of the parameters characterizing the polyphenolic fraction of the wines, analyzed after two (**a**) and twelve months (**b**) of bottle storage. Different letters mark significant differences according to ANOVA and Tukey HSD test (*p* < 0.05). Refer to Table 1 for sample codes.

**(a)**	**Untreated**				**ENZ**				**PEF 2**				**PEF 10**				**PEF 20**			
IC ^1^	3.2	±	0	b	3.7	±	0	c	2.6	±	0	a	4.7	±	0	d	5.1	±	0	e
CH ^2^	0.61	±	0	b	0.55	±	0	a	0.59	±	0	b	0.61	±	0	b	0.59	±	0	b
TPI ^3^	17.4	±	1.3	b	17.4	±	2.1	b	11.7	±	2.4	a	23.6	±	2.3	c	40.8	±	3.9	d
Anthocyanins ^4^ (mg L^−1^)	166	±	3	b	187	±	3	c	125	±	5	a	219	±	3	d	225	±	0	d
Total tannins (g L^−1^)	1.4	±	0	b	1.8	±	0.1	c	1.2	±	0	a	2.3	±	0.1	d	2.3	±	0	d
IHCl ^5^	3	±	1	a	24	±	6	b	1	±	1	a	25	±	0	b	19	±	6	b
IEtOH ^6^	0	±	0	a	0	±	0	a	0	±	1	ab	2	±	1	b	7	±	1	c
IPP ^7^	43	±	1	a	44	±	1	a	46	±	1	a	52	±	6	b	46	±	2	a
**(b)**	**Untreated**				**ENZ**				**PEF 2**				**PEF 10**				**PEF 20**			
IC	3.1	±	0.1	ab	3.5	±	0	b	2.7	±	0.4	a	4.1	±	0.1	c	4.3	±	0	c
CH	0.77	±	0.01	a	0.78	±	0	a	0.82	±	0	b	0.77	±	0.01	a	0.79	±	0	ab
TPI	31.7	±	2.9	ab	36.8	±	1.5	bc	24.6	±	1.6	a	43.6	±	3.8	cd	44.8	±	2.8	d
Anthocyanins ^1^ (mg L^−1^)	52	±	5	a	59	±	5	a	48	±	0	a	54	±	10	a	78	±	5	b
Total tannins (g L^−1^)	1.6	±	0.1	ab	1.8	±	0.3	bc	1.2	±	0.1	a	2.2	±	0.2	cd	2.4	±	0.2	d
IHCl	37	±	4	b	22	±	3	ab	19	±	10	ab	21	±	7	ab	13	±	11	a
IEtOH	*n.d.* ^8^				*n.d.*				*n.d.*				*n.d.*				*n.d.*			
IPP	80	±	6	b	81	±	3	b	69	±	3	a	85	±	2	b	79	±	4	ab

^1^ IC: color intensity; ^2^ CH: color hue; ^3^ TPI: total phenolic index; ^4^ Spectrophotometric determination; ^5^ IHCl: hydrochloric acid index; ^6^ IEtOH: ethanol index; ^7^ IPP: index of polymerized pigments; ^8^ *n.d.:* not detectable.

**Table 4:** Results of the HPLC separation of anthocyanins in the experimental wines after two (**a**) and twelve months (**b**) of bottle storage. The data reported are absolute areas/1000 (means ± SD). Different letters mark significant differences according to ANOVA and Tukey HSD test (*p* < 0.05). Refer to Table 1 for sample codes.

**(a)**	**Untreated**				**ENZ**				**PEF 2**				**PEF 10**				**PEF 20**			
D3G	356	±	12	b	498	±	19	c	286	±	5	a	545	±	5	d	635	±	10	e
C3G	39	±	4	b	45	±	4	b	23	±	0	a	56	±	1	c	55	±	1	c
Pt3G	776	±	1	b	945	±	41	c	640	±	11	a	1070	±	18	d	1222	±	13	e
Peo3G	1194	±	13	b	1413	±	18	c	848	±	9	a	1747	±	76	d	1686	±	37	d
M3G	11716	±	134	b	12383	±	43	c	9602	±	98	a	14085	±	36	d	14144	±	94	d
Vit B	*n.d.* ^1^				*n.d.*				*n.d.*				*n.d.*				*n.d.*			
Vit A	173	±	15	a	206	±	12	b	152	±	6	a	248	±	6	c	242	±	5	c
M3GAc	295	±	26	b	275	±	6	b	188	±	7	a	311	±	25	bc	342	±	4	c
M3GpCm	1828	±	175	ab	2245	±	190	bc	1491	±	180	a	2350	±	244	bc	2556	±	187	c
**(b)**	**Untreated**				**ENZ**				**PEF 2**				**PEF 10**				**PEF 20**			
D3G	43	±	3	b	23	±	9	a	28	±	7	a	85	±	5	c	29	±	3	ab
C3G	*n.d.*				*n.d.*				*n.d.*				*n.d.*				*n.d.*			
Pt3G	73	±	6	b	29	±	13	a	50	±	10	ab	126	±	8	c	34	±	0	a
Peo3G	118	±	12	b	26	±	12	a	46	±	11	a	175	±	10	c	33	±	2	a
M3G	1235	±	101	c	407	±	155	a	793	±	134	b	1813	±	115	d	481	±	7	a
Vit B	*n.d.*				*n.d.*				*n.d.*				*n.d.*				*n.d.*			
Vit A	262	±	0	c	233	±	0	b	188	±	3	a	332	±	1	e	317	±	3	d
M3GAc	26	±	1	a	22	±	6	a	20	±	4	a	36	±	2	b	25	±	1	a
M3GpCm	66	±	2	b	20	±	8	a	37	±	12	a	144	±	18	c	25	±	2	a

Detected anthocyanins were: delphinidin-3-*O*-glucoside (D3G), cyanidin-3-*O*-glucoside (C3G), petunidin-3-*O*-glucoside (Pt3G), peonidin-3-*O*-glucoside (Peo3G), malvidin-3-*O*-glucoside (M3G), vitisin B (Vit B), vitisin (Vit A), malvidin-3-*O*-(6-acetyl)-glucoside (M3GAc), malvidin-3-*O*-(6-*p*-coumaroyl)-glucoside (M3GpCm. ^1^ *n.d.*: not detected.

**Table 5:** Results of the sensory evaluation carried out on experimental wines after two (**a**) and twelve months (**b**) of bottle storage. The data reported are the sums of the ranks calculated for each sample after the ranking test. The lower the sum of the ranks calculated for a sample, the higher the intensity perceived by the panel for the attribute considered. Different letters mark significant differences according to the Friedman test (*p* < 0.05). Refer to Table 1 for sample codes.

**(a)**	**Color** **Intensity**		**Orange** **Hue**		**Fruity** **Aroma**		**Vegetal,** **Herbaceous**		**Body,** **Structure**		**Astringency**		**Global** **Impression**	
Untreated	25	bc	12	a	21	a	12	a	18	ab	18	a	17	a
ENZ	17	abc	21	ab	19	a	19	a	20	ab	16	a	16	a
PEF 2	29	c	10	a	17	a	21	a	27	b	23	a	23	a
PEF 10	6	a	28	b	18	a	18	a	11	a	16	a	17	a
PEF 20	13	ab	19	ab	15	a	20	a	14	ab	17	a	17	a
**(b)**	**Color** **Intensity**		**Orange** **Hue**		**Fruity** **Aroma**		**Vegetal,** **Herbaceous**		**Body,** **Structure**		**Astringency**		**Global** **Impression**	
Untreated	25	ab	16	ab	20	a	23	a	20	a	21	a	15	a
ENZ	23	ab	18	ab	22	a	16	a	21	a	23	a	23	ab
PEF 2	31	b	13	a	26	a	18	a	28	a	29	a	31	b
PEF 10	15	a	29	b	20	a	26	a	17	a	18	a	18	ab
PEF 20	11	a	29	b	17	a	22	a	19	a	14	a	18	ab

## Supplementary Materials

The following are available online at https://www.mdpi.com/2304-8158/9/4/414/s1, Figure S1. Samples on the third day of maceration, showing the greater breakdown of the skin integrity, for the treatments with a higher specific energy.
