# Supplementary material for: Pulsed Electric Field Processing of Red Grapes (cv. Rondinella): Modifications of Phenolic Fraction and Effects on Wine Evolution
Source: Foods. 2020 Apr 2;9(4):414. doi: 10.3390/foods9040414 (PMC7230476; doi:10.3390/foods9040414)
Supplement: Supplementary file 1 [file foods-09-00414-s001.pdf]

Supplementary Material

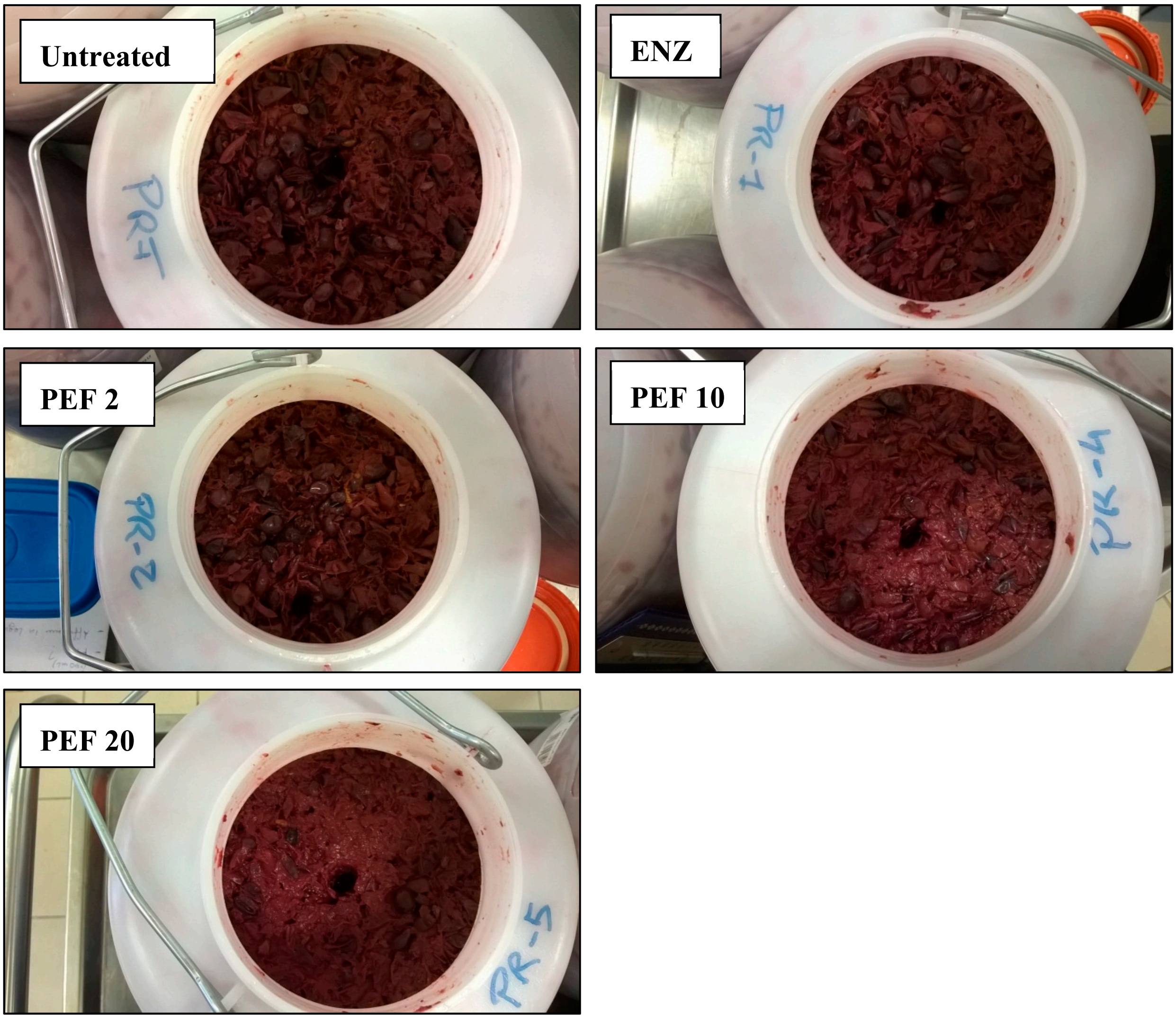

Figure S1. Samples at the third day of maceration, showing the greater breakdown of the skin integrity, for the treatments with higher specific energy. Pictures taken before punching-down the pomace. Untreated: control, no treatment; ENZ: pectolytic enzymes (20 mg kg^{-1}); PEF 2: PEF treatment, 2 kJ kg^{-1}; PEF 10: PEF treatment, 10 kJ kg^{-1}; PEF 20: PEF treatment, 20 kJ kg^{-1}. PEF, pulsed electric field.
